# Descemet Membrane Endothelial Keratoplasty with Irregular-Edged Graft: A Salvage Method for Large Radial Graft Tears

**DOI:** 10.4274/tjo.39019

**Published:** 2018-04-25

**Authors:** Mehmet Cüneyt Özmen, Nilay Dilekmen, Erdem Yüksel, Bahri Aydın, Fikret Akata

**Affiliations:** 1 Gazi University Faculty of Medicine, Department of Ophthalmology, Ankara, Turkey; 2 Bingöl State Hospital, Ophthalmology Clinic, Bingöl, Turkey; 3 Private Practice

**Keywords:** Descemet membrane endothelial keratoplasty, large radial tears, irregular-edged Descemet membrane graft

## Abstract

Large radial tears of donor Descemet membrane (DM) during the preparation of Descemet membrane endothelial keratoplasty (DMEK) grafts can make the trephination stage impossible because of small graft diameter. This results in irregular-edged grafts. In this study, we report two pseudophakic bullous keratopathy patients who underwent DMEK surgery with irregular-edged Descemet membrane (DM) grafts. Main outcome measures were preoperative and postoperative 1-, 3-, and 6-month best corrected visual acuity (BCVA), endothelial cell density (ECD) and central corneal thickness (CCT). Intraoperative and early postoperative complications were also evaluated. Both irregular-edged grafts were successfully implanted into the anterior chamber, unfolded, and attached to the posterior corneal stroma. Patients’ BCVA at 6 months was 1.0 (Snellen equivalent: 20/20) and 0.6 (Snellen equivalent: 20/32) respectively. Decrease in ECD at the last visit was 27% and 25%. CCT decreased from 723 μm and 850 μm to 530 μm and 523 μm, respectively. No intraoperative complications occurred except for the large radial Descemet membrane graft tears that developed during donor DM stripping. None of the cases needed a rebubbling procedure postoperatively. We have demonstrated that irregular-edged DM grafts can be successfully implanted for DMEK surgery with good clinical outcomes.

## Introduction

Descemet membrane endothelial keratoplasty (DMEK) is the latest refinement of endothelial keratoplasty procedures. Providing an exact anatomical replacement of only what is removed, it gives the possibility of excellent visual acuity with shorter healing time as well as minimal risk of immunological rejection.^1,2^ However, preparing the 15-µm-thick Descemet membrane (DM) graft is still a challenging issue and is sometimes complicated by surgeon- or donor-related DM graft tears and graft failure. Standardized techniques for graft preparation, surgical instruments designed for endothelial keratoplasty, and accumulating experience over time have led to a significant reduction of tissue loss. However, as the donor-related risk factors for failure in donor tissue preparation have not been clearly determined, the potential risk for radial DM tearing still exists. By pulling the torn flaps peripherally and skipping the trephination stage of the graft preparation technique described by Melles et al.,^3,4^ these irregular-edged grafts can still be successfully implanted. In this study, we describe the clinical results of two eyes with pseudophakic bullous keratopathy treated with DMEK with irregular-edged DM graft.

## Case Report

### Patients

Case 1 was a 72-year-old female patient with a 1-year history of pseudophakic bullous keratopathy in the right eye. Intraocular lens was in the bag and posterior capsule was intact. Best corrected visual acuity (BCVA) was counting fingers from 1 meter (20/2000) with a central corneal thickness (CCT) of 723 µm. Case 2 was a 48-year-old female patient who underwent primary suturation after a penetrating corneal injury to her left eye 2 years earlier. She had scleral fixation of an IOL and trabeculectomy for glaucoma 1.5 years earlier. She had endothelial decompensation in the left eye with a BCVA of hand motions (20/20000). Her CCT was 850 µm with a central corneal scar. Both cases were contact lens-dependent and were using 5% hypertonic ophthalmic solutions.

### Donors and Graft Preparation

Donor cornea-scleral buttons were obtained less than 24 h postmortem (donor ages 50 and 67 years, endothelial cell density (ECD) 2450 and 2530 cells/mm^2^, respectively) and stored in corneal storage medium (Optisol-GS, Bausch & Lomb, Irvine, CA, USA) at 4 °C. Graft preparation was done preoperatively in the operating room by applying the technique described in detail by Melles et al.^3,4^ Cornea-scleral buttons were mounted endothelial side up on a holder. Descemet membrane–endothelial complex (DEC) was dissected gradually from the periphery to the center starting with a hockey stick knife. In both cases, large radial tears formed during stripping because of focal adhesions between DM and stroma. Radial tears were manipulated by pulling the flap peripherally, forming an irregular edge with no radial tears. This technique allowed preparation of irregular-edged, non-uniform grafts. In our standard technique, stripped DEC surrounded by a 360-degree trabecular meshwork ring is transferred on a soft contact lens and trephined to obtain a 9-9.5-mm regular-edged circular graft. However, since both grafts were smaller than 9 mm in the largest dimension, trephination was not possible. For both cases, the irregular-edged grafts were formed into a DEC roll with the endothelium on the outside and stored in saline until the recipient’s cornea was prepared.

### Surgical Technique

**Figure 1:**
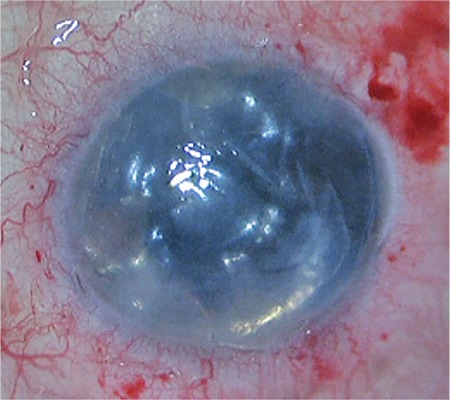
Graft position of case 1 at the end of surgery

Both surgeries were performed under general anesthesia using the “no-touch” technique described previously with slight modifications.^1,5^ After making three 23-gauge and one 3.2-mm keratotomies, the DM was scored at a radius of 9.5 mm with a reverse Sinskey hook (DORC international BV, Holland) and removed from the anterior chamber under complete air fill. Then 0.1 mL of acetylcholine chloride (Miochol-e, 20 mg/mL, Novartis) was used to induce myosis in case 2 to reduce the risk of posterior dislocation of DEC roll. The DEC roll was injected into the recipient’s anterior chamber using a glass injector (DORC international BV, Holland) and oriented endothelial side down by indirect manipulations with air and balanced salt solution. The graft was then uncurled and centered with a combination of corneal dome compression and sweeping of the corneal surface with cannulas. We ensured the graft covered the visual axis. Once the graft was centered, an air bubble was injected underneath the graft and left for 30 minutes under complete air fill. At the end of the procedure, 75% air fill was left (Figure 1). Incisions were not sutured.

Case 1 underwent a surgical peripheral iridotomy intraoperatively, while Case 2 had a preexisting peripheral iridectomy due to previous trabeculectomy surgery. The patients were requested to lie in supine position for 12 hours after surgery. We recommended 1% prednisolone acetate drops 5 times daily for 8 weeks (then tapered gradually), and 0.5% moxifloxacin drops 5 times daily for 2 weeks.

### Postoperative Evaluations

**Figure 2:**
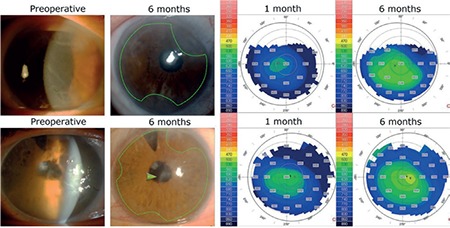
Preoperative and postoperative 6-month photos and corneal thickness maps of patients. Green lines outline the approximate position of irregular-edged Descemet membrane endothelial keratoplasty graft. Green arrowhead shows corneal scar in case 2 (First row represents case 1 and second row represents case 2)

The postoperative period was uneventful for both patients, with no DEC detachments requiring rebubbling. Both eyes had improved corneal clarity (Figure 2) and increased BCVA at 6 months. BCVA at 1 month and 6 months was 0.5 and 1.0 (with a -3.0 D cylinder) for case 1, and 0.2 and 0.6 (with a -2.0 D cylinder) for case 2, respectively. Intraocular pressures of cases 1 and 2 at 6 months were 18 mmHg and 20 mmHg, respectively.

Decrease in endothelial cell density at 6 months was 27% and 25% respectively. Central corneal thickness decreased from 723 µm and 850 µm before surgery to 530 µm and 523 µm respectively at postoperative 6 months (Figure 2). Preoperative and postoperative BCVA, ECD, and CCT values are summarized in Table 1.

In the immediate postoperative period, both cases had focal corneal edema limited to the areas of bare corneal stroma. These edematous areas corresponded with the denuded recipient stroma with no DEC because of the irregular graft shape. Edema resolved by the postoperative 6-month visit.

## Discussion

DM graft preparation for DMEK surgery has been standardized previously with very low rates of tissue damage due to preparation.^6,7^ However it can be complicated with large radial tears, making the trephination and usage of DEC graft impossible. Especially if the graft is prepared by the surgeon prior to surgery in the operating room, radial tear risk increases due to time limitations and surgical stress. To reduce the surgical stress, the surgeon can use eye bank-prepared donor tissue or prepare the tissue days before the surgery. It has been shown that eye bank-prepared grafts and surgeon-prepared grafts do not differ in terms of graft survival outcomes. Both preparation methods have a 5% graft preparation failure rate due to strong adhesions between DEC and stroma.^8^ Although grafts prepared by the eye bank might have an advantage in decreasing the surgical stress, eye bank-preparation is more expensive compared to preoperative preparation. Even when the surgeon is preparing the graft days before surgery, the use of an extra corneal storage solution increases the total cost of the procedure. Radial tears in DEC grafts that are formed during preparation might increase in size during implantation and unfolding of the graft, resulting in dehiscence of the graft postoperatively. 

This complication can be managed with a modification of standardized donor tissue preparation technique: rescuing the radial tears by pulling the flap peripherally, skipping the trephination phase, and implanting the irregular-edged graft. Recently, two studies have shown that partial DEC grafts can be implanted and may yield good clinical outcomes.^9,10^ Since the risk of losing tissue is still the biggest concern of DMEK graft preparation, the modification we propose can be a salvage method for using grafts with large radial tears. 

With irregular-edged grafts, large areas of denuded stroma with edema can be seen in the first postoperative months. Spontaneous resolution of focal edematous areas without DEC may be attributed to the migration of donor and/or recipient endothelial cells onto the denuded stroma.^9,10,11^

Although two cases may not be enough to evaluate the potential clinical outcomes of irregular-edged grafts, our results seem promising to salvage grafts with large radial tears. These irregular-edged, non-uniform DM grafts might be successfully implanted for DMEK surgery with the potential for favorable clinical outcomes. 

## Figures and Tables

**Table 1:**
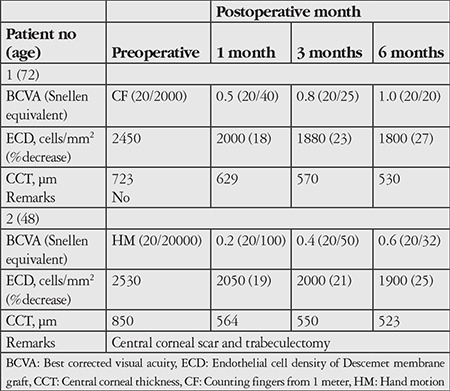
Outcomes following Descemet membrane endothelial keratoplasty with irregular edged grafts
